# Long-Term Athletic Development in Youth Alpine Ski Racing: The Effect of Physical Fitness, Ski Racing Technique, Anthropometrics and Biological Maturity Status on Injuries

**DOI:** 10.3389/fphys.2017.00656

**Published:** 2017-08-31

**Authors:** Lisa Müller, Carolin Hildebrandt, Erich Müller, Christian Fink, Christian Raschner

**Affiliations:** ^1^Department of Sport Science, University of Innsbruck Innsbruck, Austria; ^2^Department of Sport Science and Kinesiology, University of Salzburg Salzburg, Austria; ^3^Research Unit for Orthopedic Sports Medicine and Injury Prevention, Institute of Psychology (ISAG), The Health & Life Sciences University (UMIT) Hall, Austria; ^4^Gelenkpunkt – Sports and Joint Surgery Innsbruck, Austria

**Keywords:** injury risk factors, physical fitness, racing technique, biological maturity status, anthropometrics, youth alpine ski racing, neuromuscular training

## Abstract

Alpine ski racing is known to be a sport with a high risk of injuries. Because most studies have focused mainly on top-level athletes and on traumatic injuries, limited research exists about injury risk factors among youth ski racers. The aim of this study was to determine the intrinsic risk factors (anthropometrics, biological maturity, physical fitness, racing technique) for injury among youth alpine ski racers. Study participants were 81 youth ski racers attending a ski boarding school (50 males, 31 females; 9–14 years). A prospective longitudinal cohort design was used to monitor sports-related risk factors over two seasons and traumatic (TI) and overuse injuries (OI). At the beginning of the study, anthropometric characteristics (body height, body weight, sitting height, body mass index); biological maturity [status age at peak height velocity (APHV)]; physical performance parameters related to jump coordination, maximal leg and core strength, explosive and reactive strength, balance and endurance; and ski racing technique were assessed. Z score transformations normalized the age groups. Multivariate binary logistic regression (dependent variable: injury yes/no) and multivariate linear regression analyses (dependent variable: injury severity in total days of absence from training) were calculated. *T*-tests and multivariate analyses of variance were used to reveal differences between injured and non-injured athletes and between injury severity groups. The level of significance was set to *p* < 0.05. Relatively low rates of injuries were reported for both traumatic (0.63 TI/athlete) and overuse injuries (0.21 OI/athlete). Athletes with higher body weight, body height, and sitting height; lower APHV values; better core flexion strength; smaller core flexion:extension strength ratio; shorter drop jump contact time; and higher drop jump reactive strength index were at a lower injury risk or more vulnerable for fewer days of absence from training. However, significant differences between injured and non-injured athletes were only observed with respect to the drop jump reactive strength index. Regular documentation of anthropometric characteristics, biological maturity and physical fitness parameters is crucial to help to prevent injury in youth ski racing. The present findings suggest that neuromuscular training should be incorporated into the training regimen of youth ski racers to prevent injuries.

## Introduction

Alpine ski racing is a physically demanding sport and presents a high risk of injury independent of age and gender (Neumayr et al., 2003; Spörri et al., 2017). In elite alpine ski racing, injury rates of more than 36 injuries/100 athletes have been reported, 36% being severe and partly career ending (Bere et al., 2013; Spörri et al., 2017). The most common injuries were ligament injuries in the knee (35.6%), with the rupture of the anterior cruciate ligament (ACL) being the most frequent diagnosis, representing 13.6% of all injuries in World Cup athletes (Flørenes et al., 2009). To date, most studies have concentrated on elite alpine ski racers. Determining the implications for prepubescent athletes still remains a challenge, despite providing the basis for long-term athletic development (Lloyd et al., 2015; Spörri et al., 2017). The popularity of alpine ski racing in Austria is reflected in the high number of specialized ski boarding schools. However, sport specialization at a young age should not negatively affect skeletally immature athletes (Maffulli et al., 2010). Therefore, epidemiological studies with respect to injuries, and in a further step, studies investigating the injury risk factors in youth ski racing are particularly important as a first and second step of the injury prevention sequence (Van Mechelen et al., 1992).

To date, only two epidemiological studies have been conducted at the adolescent level with ski racers older than 15 years of age (Hildebrandt and Raschner, 2013; Westin et al., 2013) and one study at the youth level among athletes younger than 15 years of age (Müller et al., 2017). Westin et al. (2013) obtained similar results compared with the World Cup level with respect to injury incidence (1.7 injuries/1000 ski hours), severity (49% severe) and the most-affected body part (knee: 41%) among Swedish adolescent ski racers (>16 years). Recently, Müller et al. (2017) observed a relatively low incidence of traumatic injuries (0.86 traumatic injuries/1,000 h of training) in a cohort of ski racers younger than 15 years of age.

In addition to traumatic injuries, overuse injuries represent a problem among adolescent ski racers. Hildebrandt and Raschner (2013) investigated 15–19 year old adolescent ski racers of a ski boarding school over two consecutive school years (September-July). More than half of the investigated athletes had at least one overuse injury during the two-season study period, most of which were low back pain (Hildebrandt and Raschner, 2013). The rate of overuse injuries among ski racers younger than 15 years was much lower (0.21 overuse injuries/athlete) (Müller et al., 2017). However, this study was limited to an epidemiological point of view; scientific research concerning overuse injuries in general and risk factors for overuse injuries among youth ski racers in particular, is still lacking.

Furthermore, a prospective data analysis of skiing-specific risk factors for injuries in youth ski racers who specialize at an early age is also lacking. Previous research has indicated poor physical fitness in youth athletes as a risk factor for sports-related injuries (Carter and Micheli, 2011). Regarding alpine ski racing, Raschner et al. (2012) revealed that insufficient core strength or core strength imbalance was a critical factor for sustaining an ACL injury in adolescent ski racers (15–19 years). However, no data exist about the level of physical fitness of prepubescent athletes and the risk of sustaining an injury, indicating the need for additional data pertaining to possible risk factors for overuse injuries among this youngest age group.

In this context, injury prevention should also include the consideration of biological maturity status because late maturing athletes might be at a higher risk for both overuse and traumatic injuries. It is well known that growth-related factors such as biological immaturity contribute to overuse injuries (DiFiori et al., 2014). The risk of sustaining such an overuse injury is strongly intensified during the adolescent growth spurt (Fort-Vanmeerhaeghe et al., 2016). Additionally, studies of soccer players revealed that late maturing athletes were at a higher risk for overuse injuries (Van der Sluis et al., 2015) or severe traumatic injuries (Le Gall et al., 2007). To date, however, the biological maturity status has not been assessed as a possible risk factor for injuries in youth ski racing.

Furthermore, Spörri et al. (2017) showed in their review article that technical mistakes represent a risk factor for injuries in World Cup alpine ski racers. Training of a stable “skiing technique” in combination with specific neuromuscular training was suggested as a potential preventive measure within this context (Spörri et al., 2017). The combination of frontal bending, lateral bending, and torsion in the loaded trunk has been found to be a potential risk factor for overuse injuries in elite ski racers (Spörri et al., 2015); showing that the ski racing technique has to be well established in order to minimize the load on the trunk in such situations. However, thus far, skiing-technique has not been investigated as a possible risk factor in youth ski racing.

Overall, a suitable screening strategy starting at a young age should focus on the identification of modifiable risk factors (Lloyd et al., 2015). In support of this, Spörri et al. (2017) clearly demonstrated the need for (and lack of) monitoring and preventing injuries at the youth level, and the importance of being able to identify the possible risk factors. Therefore, the aim of the present study was to investigate intrinsic risk factors for injuries such as anthropometrics, biological maturity status, physical fitness and ski racing technique among youth ski racers younger than 15 years of age.

## Methods

### Study design

A two-season prospective study design was used to record injuries and possible risk factors in a cohort of elite youth alpine ski racers younger than 15 years of age. s. An internet-based database (training data base, injury data base) was developed to record all training data and injuries. The coaches recorded all training data including the presence or absence (due to injury) of the athletes immediately after each skiing specific and athletic specific training session. One member of the study team registered all injury data on a weekly basis. The study was performed according to the Declaration of Helsinki and was approved by the Institutional Review Board of the Department of Sport Science of the University of Innsbruck as well as the Board for Ethical Questions of the University of Innsbruck. The athletes and their parents were informed of the study aims, procedures and risks before written informed consent was obtained.

### Participants

The study was performed in cooperation with a highly regarded skiing specific secondary modern school in Austria. Participants of the study were pupils of this ski boarding school, aged 9–14 years. Eighty-five athletes who were free of injury at the beginning of the study were enrolled.

### Data collection

#### Injury registration

Using the training data base of the ski boarding school, coaches recorded all relevant training data immediately after each training session (skiing specific: technique, race; athletic specific: endurance, neuromuscular, strength training) including among others duration, volume, intensity, contents, presence/absence of athletes due to injury. Exposure time was recorded for each athlete as the number of minutes of all training sessions (skiing- and athletic-specific training). The data entry was user-friendly, because all necessary information was limitative and the coaches only had to mark the appropriate boxes by ticking. If an athlete was absent, the coaches indicated the cause (illness, traumatic injury, overuse injury, others) and an automatic mail was sent to the study team.

One member of the study team (sports scientist and/or physician) then contacted the coaches, physiotherapists and/or physicians of the ski boarding school to get detailed information about the injury. Then all injuries (traumatic and overuse) that occurred in skiing-specific or athletic-specific training sessions, as well as in competitions, and that caused absence from training for at least 1 day, were registered. All data were systematically checked with the coaches, physiotherapists and physician either face-to-face or by telephone. In case of injuries that required medical attention, a detailed medical report was provided. Further details with respect to injuries that occurred and study design are presented in Müller et al. (2017).

A traumatic injury was defined as an injury with a sudden onset based on time-loss definition (Brooks and Fuller, 2006), and the type of traumatic injury, as well as the affected body part were defined according to the injury surveillance consensus paper of the International Olympic Committee (Junge et al., 2008). Injury severity was classified according to Fuller et al. (2006). An injury was classified as minimal with a time loss of 1–3 days, as mild (4–7 days), moderate (8–28 days), severe (>28 days) or career ending (Fuller et al., 2006). Additionally, the mean injury severity of each athlete was calculated (total days of absence/total number of injuries). An overuse injury was defined as any physical complaint without a single identifiable event being responsible (Clarsen et al., 2013). With respect to young athletes, overuse injuries include apophyseal injuries and physeal stress injuries (DiFiori et al., 2014).

#### Anthropometrics and biological maturity

The anthropometric characteristics body height (cm), body weight (kg), leg length (cm), sitting height (cm), and body mass index (BMI; m^2^/kg) of the athletes were assessed at the beginning of the study (September). Biological maturity status was assessed by the non-invasive method of calculating the age at peak height velocity (APHV) using gender-specific prediction equations (Mirwald et al., 2002). The validity of this method was previously proven for youth ski racers of the same age (Müller et al., 2015). As proposed by Sherar et al. (2007), the athletes were divided into three maturity groups (early, normal or late maturing) based on the mean (M) ± standard deviation (SD) of the APHV of the total sample (separated by gender; normal: APHV within M ± SD; early: APHV <M-SD; late: APHV>M+SD). The percentiles of body height, body weight and body mass index were classified according to Braegger et al. (2011). Standardized percentile curves of BMI, body weight and height were used to classify each athlete into three categories: <25% percentile = below average, 25–75% percentile = average, >75% percentile = above average.

#### Physical fitness testing

The physical fitness parameters were tested in the sports laboratory at the Department of Sport Science prior to the start of the season (September). Results of the following tests were included: postural stability test (S3-Check), agility test (jump coordination test including 26 jumps), isometric leg extension strength test (unilateral leg press), isometric core strength test (Back-Check, Dr. Wolff Sports & Prevention GmbH, Arnsberg, Germany), power test (Counter Movement Jump, Kistler force plate), reactive strength test (Drop Jump, Kistler force plate) and aerobic endurance test (12-min Cooper test). A detailed description of the testing procedures, materials and test-retest reliability can be found in the studies of Raschner et al. (2012, 2013). Table 1 shows the appropriate parameters and abbreviations for each test. All tests were performed in a standardized order. Three attempts were allowed for each test, and only the best result was used for the analysis. The values obtained for the athletes were then classified into age- and gender-specific norm data, which were calculated (mean ± ½ standard deviation) based on a comprehensive, sport-specific data pool starting in 1996 (Raschner et al., 2013). Based on their results, the young ski racers were categorized into three groups (average, above and below average).

**Table 1:** Physical fitness parameters.

**Abbreviation**	**Test**	**Parameters**	**Category**
JCT	Jump coordination test	Time in s	Coordination
ULST	Unilateral leg press strength test	Relative leg strength (REL LS) in N/kg left and right total	Maximal strength
		Ratio relative left/right leg strength (L:R LS)	
CST	Core strength test	Relative flexion strength (REL FS) in N/kg	Maximal strength
		Relative extension strength (REL ES) in N/kg	
		Ratio relative flexion/extension strength (FLE:EXT S)	
CMJ	Counter movement jump	Jump height in cm	Explosive strength
DJ	Drop jump	Reactive strength index (RSI) in mm/ms	Reactive strength
ST	Stability test (S3 Check)	Stability index (left-right index)	Balance
CT	Cooper test	Total distance in m	Endurance

#### Ski racing technique

The athletes had to pass an entrance exam before they were allowed to frequent the ski boarding school. The entrance exam consisted of skiing-specific exercises and physical performance tests. Among other factors, ski racing technique in three different racing situations (slalom, giant slalom, combi race) was evaluated by three independent experts in youth ski racing according to the Austrian school grading scale (1 = perfect; 5 = failed). Each expert rated the performance of all athletes by a blinded procedure. The mean of the grades of the three experts and the three racing conditions were calculated and considered in the analyses.

### Statistical analyses

Descriptive statistics are presented as the M ± SD for continuous variables and as frequency counts and percentages for categorical variables. The rates of traumatic and overuse injuries per athlete were calculated as number of injured ski racers divided by the total number. The normal distribution was tested using Kolmogorov-Smirnov tests. Two regression analyses were performed. With respect to occurrence of injury (traumatic and overuse injury combined), a binary logistic regression analysis (backward LR method; dependent variable: injury yes/no) was performed. Regarding injury severity, a multiple linear regression analysis with stepwise backward elimination was performed (dependent variable: days of absence from training due to injury). A gender- and age-specific z-transformation was performed for all variables (Raschner et al., 2012), except for ratios, APHV, BMI and racing technique. The independent variables for the linear regression analyses were the anthropometric characteristics (BMI; z values of height, weight, sitting height, leg length), APHV as an indicator of biological maturity status, the *z*-values of the physical performance test results (see Table **3**) and the indicator of ski racing technique (mean of grades). The included independent variables were previously tested for collinearity; no collinearity was present. Nagelkerke's R^2^ was calculated to determine the power of the model used. Independent *t*-tests were used to assess differences between injured and non-injured athletes with respect to the significant variables of the binary logistic regression analysis. The number of days absent from training per injury (in mean) were additionally calculated to categorize the athletes' mean injury severity (1–7 days: minimal-mild; 8–28 days: moderate; >28 days severe). Multivariate analyses of variance with Bonferroni alpha adjustment were performed (dependent variables: significant variables of regression analyses concerning injury severity; independent variable: categories of severity per injury in mean; contrast: Helmert; *post-hoc*: Scheffé) to assess differences in the significant injury risk factors (assessed by the linear regression analysis) between the three categories of injury severity. The level of significance was set to *p* < 0.05. All calculations were performed using IBM SPSS 23.0 (IBM Corporation, Armonk, NY, USA).

## Results

### Participants

Over the 2-year study period, three athletes dropped out of school and one athlete had missing values in the physical performance tests; thus, 81 athletes (50 males, 31 females; 11.6 ± 1.4 years) were included in the analyses. The anthropometric data of the 81 participants separated by gender are presented in Table 2. The percentiles of body height, weight and BMI are presented according to the three groups of biological maturity status in Table 3.

**Table 2:** Anthropometric data at the start of the study according to gender.

	**Male (*n* = 50)**	**Female (*n* = 31)**
	**M ± SD**	**M ± SD**
Age [yrs]	11.5 ± 1.5	11.8 ± 1.3
Height [cm]	153.3 ± 9.0	154.9 ± 7.7
Weight [cm]	42.3 ± 8.2	43.8 ± 7.9
BMI [kg/m^2^]	17.8 ± 1.8	18.1 ± 2.2
APHV [years]^*^	13.7 ± 0.5	12.0 ± 0.5

* *Significant gender specific difference [t_(79)_ = 13.856; p < 0.001]*.

*BMI, body mass index; APHV, age at peak height velocity*.

**Table 3:** Percentiles of anthropometric characteristics according to biological maturity status.

	**Early (*n* = 10)**	**Normal (*n* = 61)**	**Late (*n* = 10)**
	**M [95% CI]**	**M [95% CI]**	**M [95% CI]**
Percentile height	71.1 [55.7-84.8]	45.3 [40.8-49.6]	23.6 [15.0-34.2]
Percentile weight	64.2 [49.1-78.0]	41.8 [36.5-46.7]	22.9 [14.2-33.2]
Percentile BMI	53.5 [38.4-69.9]	42.2 [35.6-47.8]	30.7 [19.9-42.3]

### Rate of injuries

In total, 69 injuries (traumatic and overuse) were recorded from 40 athletes (49.4%; 14 females, 26 males). Traumatic injuries were reported from 34 athletes (42.0%; 14 females, 20 males), representing a rate of 0.63 traumatic injuries/athlete. Nearly half of the traumatic injuries affected the bones and the knee was the most common injured body part (>1/3 of the injuries). Seventeen overuse injuries were described from 13 athletes (16.0%; 2 females, 11 males), representing a rate of 0.21 overuse injuries / athlete. Most of the overuse injuries comprised muscle and tendon structures and similar to the traumatic injuries, the knee was the most affected body part. Further details of the injuries that occurred are presented in Müller et al. (2017). During the two-season study period, 41 athletes (50.6%; 17 females, 24 males) sustained neither an overuse nor a traumatic injury.

### Injury risk factors

The percentages of injured and non-injured athletes (traumatic injuries, overuse injuries and injuries in total) are presented in Table 4 according to biological maturity (normal, early and late maturing) and anthropometrics (percentiles of body height, body weight and BMI).

**Table 4:** Affected and non-affected athletes of traumatic and overuse injuries with respect to biological maturity status and anthropometrics.

		**Traumatic injuries (*****n*** = **34)**		**Overuse injuries (*****n*** = **13)**		**Overall injuries (*****n*** = **40)**	
		**Yes [%]**	**No [%]**	**Yes [%]**	**No [%]**	**Yes [%]**	**No [%]**
Biological maturity status	Early (*n* = 10)	30.0	70.0	30.0	70.0	50.0	50.0
	Normal (*n* = 61)	42.6	57.4	11.5	88.5	47.5	52.5
	Late (*n* = 10)	50.0	50.0	30.0	70.0	60.0	40.0
Percentile body height	Above average (*n* = 12)	25.0	75.0	8.3	91.7	33.3	66.7
	Average (*n* = 55)	43.6	56.4	18.2	81.8	50.9	49.1
	Below average (*n* = 14)	50.0	50.0	14.3	85.7	57.1	42.9
Percentile body weight	Above average (*n* = 9)	33.3	66.7	33.3	66.7	55.6	44.4
	Average (*n* = 50)	36.0	64.0	16.0	84.0	42.0	58.0
	Below average (*n* = 22)	59.1	40.9	9.1	90.9	63.6	36.4
Percentile body mass index	Above average (*n* = 8)	37.5	62.5	25.0	75.0	50.0	50.0
	Average (*n* = 48)	41.7	58.3	18.8	81.3	50.0	50.0
	Below average (*n* = 25)	44.0	56.0	8.0	92.0	48.0	52.0

Table 5 shows the percentages of injured and non-injured athletes with respect to the physical performance test results based on norm groups.

**Table 5:** Affected and non-affected athletes of traumatic and overuse injuries with respect to norm groups in physical performance tests.

			**Traumatic injuries (*****n*** = **34)**		**Overuse injuries (*****n*** = **13)**		**Overall injuries (*****n*** = **40)**	
**Test**	**Variable**	**Norm group**	**yes [%]**	**no [%]**	**yes [%]**	**no [%]**	**yes [%]**	**no [%]**
CMJ	Jumping height [cm]	Above average (*n* = 9)	33.3	66.7	11.1	88.9	44.4	55.6
		Average (*n* = 53)	41.5	58.5	18.9	81.1	49.1	50.9
		Below average (*n* = 19)	47.4	52.6	10.5	89.5	52.6	47.4
DJ	RSI [index]	Above average (*n* = 12)	16.7	83.3	8.3	91.7	25.0	75.0
		Average (*n* = 57)	47.4	52.6	15.8	84.2	56.1	43.9
		Below average (*n* = 12)	41.7	58.3	25.0	75.0	41.7	58.3
CT	Distance [m]	Above average (*n* = 15)	40.0	60.0	13.3	86.7	40.0	60.0
		Average (*n* = 54)	42.6	57.4	16.7	83.3	51.9	48.1
		Below average (*n* = 12)	41.7	58.3	16.7	83.3	50.0	50.0
ULST	REL LS [N/kg]	Above average (*n* = 6)	33.3	66.7	33.3	66.7	50.0	50.0
		Average (*n* = 47)	40.4	59.6	14.9	85.1	48.9	51.1
		Below average (*n* = 28)	46.4	53.6	14.3	85.7	50.0	50.0
								
	L:R LS [ratio]	Norm (*n* = 55)	45.5	54.5	14.5	85.5	52.7	47.3
		Out of norm (*n* = 26)	34.6	65.4	19.2	80.8	42.3	57.7
CST	REL FS [N/kg]	Above average (*n* = 47)	38.3	61.7	14.9	85.1	46.8	53.2
		Average (*n* = 32)	50.0	50.0	18.8	81.3	56.3	43.7
		Below average (*n* = 2)	0	100	0	100	0	100
								
	REL ES [N/kg]	Above average (*n* = 10)	20.0	80.0	20.0	80.0	30.0	70.0
		Average (*n* = 35)	37.1	62.9	17.1	82.9	45.7	54.3
		Below average (*n* = 36)	52.8	47.2	13.9	86.1	58.3	41.7
								
	FLE:EXT S [ratio]	Norm (*n* = 25)	44.0	56.0	12.0	88.0	48.0	52.0
		Out of norm (*n* = 56)	41.1	58.9	17.9	82.1	50.0	50.0
ST	Stability Index [index]	Above average (*n* = 44)	45.5	54.5	11.4	88.6	75.0	25.0
		Average (*n* = 33)	39.4	60.6	15.2	84.8	42.4	57.6
		Below average (*n* = 4)	25.0	75.0	75.0	25.0	52.3	47.7
JCT	Time [s]	Above average (*n* = 18)	22.2	77.8	16.7	83.3	38.9	61.1
		Average (*n* = 50)	48.0	52.0	18.0	82.0	54.0	46.0
		Below average (*n* = 13)	46.2	53.8	7.7	92.3	46.2	53.8

Ski racing performance was considered by grading racing technique. Overall, the ski racers participated in an average of 17 (±6.3) races per season. The mean grade based on racing technique in three different racing situations (slalom, giant slalom, combi race) was 2.5 ± 0.6 (range: 1.3–3.9).

The multiple binary logistic model explained 47.3% of the variability of the athletes (Nagelkerkes *R*^2^ = 0.473); 73.5% of the cases were predicted correctly. With respect to sustaining an injury, the following variables were predictive risk factors in youth ski racers (see Table 6): body height, body weight, sitting height, core flexion strength, drop jump (DJ) contact time and DJ reactive strength index (RSI). Athletes with higher body height, higher body weight, higher sitting height, better core flexion strength, shorter DJ contact time and higher RSI were at a lower injury risk. However, independent *t*-tests revealed significant differences between injured and non-injured athletes only with respect to DJ RSI [*t*_(79)_ = 2.135; *p* = 0.036]. Non-injured athletes had a mean *z*-value of 0.18 ± 0.99, whereas injured athletes had a mean *z*-value of −0.13 ± 0.86.

**Table 6:** Regression analyses.

**Regression analysis**	**Dependent variable**	**Predictor**	***p***	**Wald/ß**
Binary logistic (*R*^2^ = 0.473)	Injury occurrence	Body height	0.003	Wald = 8.874
		Body weight	0.017	Wald = 5.680
		Sitting height	0.002	Wald = 9.699
		Core flexion strength	0.044	Wald = 4.050
		Drop jump contact time	0.024	Wald = 5.068
		Drop jump reactive strength index	0.013	Wald = 6.146
Multiple linear (*R*^2^ = 0.455)	Injury severity	Age at peak height velocity	0.011	ß = 0.373
		Ratio core flexion: extension strength	0.047	ß = −0.462
		Drop jump contact time	0.039	ß = 0.697
		Drop jump reactive strength index	0.023	ß = −1.092

### Relationship between injury risk factors and severity

**Figure 1:**
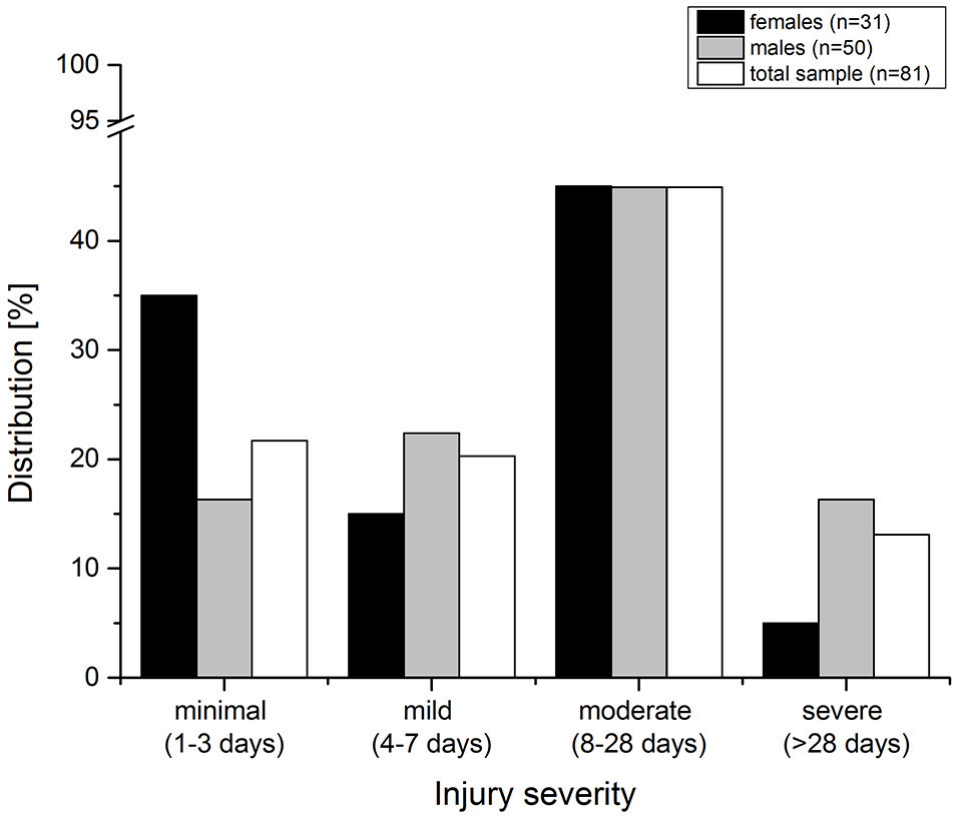
Injury severity and days of absence caused by overuse and traumatic injuries of the total sample and separated by gender.

Most injuries were classified as moderate (44.9%), leading to a time loss of training of at least 8 days. The lowest percentage of injuries was classified as severe injuries (13.1%) with over 28 days of absence from training (Figure 1); no injury was career ending. Moderate traumatic injuries accounted for 44.2%, and overuse injuries most frequently caused a loss of training, either for 8–28 days (47.1%) or more than 28 days (17.6%).

The multiple linear logistic model explained 45.5% of the variability of the athletes (Nagelkerkes *R*^2^ = 0.455). The following variables were significant predictors of injury severity (see Table 6): APHV, ratio relative flexion / extension strength (FLE:EXT S), DJ contact time and DJ RSI. Athletes with lower APHV values (which indicate that they were earlier maturing), a smaller core flexion and extension strength ratio, a shorter DJ contact time and a higher DJ RSI were more likely to miss fewer days of training due to injuries. The multivariate analyses of variance indicated a significant difference between the three groups of injury severity only with respect to DJ RSI (*F* = 3.511; *p* = 0.040). A Helmert test revealed a significant difference between the minimal-mild to moderate severity groups and the severe severity group (*p* = 0.021). Athletes with mean minimal-mild injury severity (1–7 days absence) had a mean *z*-value of 0.29 ± 0.69 with respect to DJ RSI; athletes with moderate injury severity (8–28 days) had a mean *z*-value of 0.27 ± 0.97; and athletes with severe injury severity (>28 days) had a mean *z*-value of −0.61 ± 0.70.

## Discussion

The present study supports ongoing research into modifiable risk factors among youth athletes in terms of injury prevention. It is the first study to investigate anthropometric characteristics, biological maturity status, as well as physical fitness parameters in youth alpine ski racers related to risk of traumatic or overuse injuries. The main finding was that biological maturity status, anthropometric characteristics, core strength and reactive strength represent significant injury risk factors in youth alpine ski racing. However, racing technique was not found to affect injury risk.

### Anthropometrics and biological maturity status as injury risk factors in youth ski racing

Taller, heavier and more mature athletes have been shown to have performance and selection advantages in youth alpine ski racing (Raschner et al., 1995; Müller et al., 2016). The role of anthropometric characteristics and biological maturity status as injury risk factors in youth ski racing have not been investigated, to date. In the present study, the distribution of early, normal and late maturing athletes did not differ from the expected normal distribution, in line with the study of Müller et al. (2016), in which the biological maturity status of provincial youth ski racers was normally distributed. When examining the classification of early, normal and late maturing athletes and the relationship to the percentiles of body height, body weight and BMI, it can be clearly observed that these two categorization models correspond to each other. On average, early maturing athletes had percentile values of 53.5 (BMI), 64.2 (weight), and 71.1 (height), whereas late maturing athletes had average values of 30.7 (BMI), 22.9 (weight), and 23.6(height). The values of the normal maturing athletes were in between those of the other two maturity groups. Although the validity of the easy feasible method of calculating APHV as an indicator of biological maturity status has been proven (Müller et al., 2015), the method has often been criticized. Nevertheless, the prediction equations of the APHV method include the estimation of leg length and sitting height; consequently, they consider the diverse proportions between the trunk and extremities (the long bones of the legs have a peak growth spurt before the short bones of the trunk) (Lloyd et al., 2014). Therefore, the APHV method appears to be useful in youth talent selection and injury prevention programs because it can be easily applied in a large cohort of young athletes who are not exposed to radiation (Müller et al., 2015), even though X-rays of the left wrist is still the more accurate method (Lloyd et al., 2014).

With respect to injuries, the present results revealed that the athletes who were above average in percentile body height and body weight had descriptively smaller percentages of traumatic injuries compared with the athletes who were average or below average. Additionally, early maturing athletes had descriptively lower percentages of traumatic and overuse injuries compared with normal and late maturing athletes. However, the small percentages of early and late maturing athletes must be considered. Nevertheless, the results of the regression analyses underlined the findings that anthropometric characteristics and biological maturity status play a significant role as injury risk factors in youth ski racing. With respect to occurrence of injuries, body weight, body height and sitting height were significant predictors, of which sitting height (Wald = 9.699) and body height (Wald = 8.874) seemed to have the greatest associations to injury occurrence. Concerning the severity of injuries, APHV was a significant predictor (ß = 0.373); however the association to injury severity was not that high compared to the other variables (ß = −0.462 till −1.092).

The present results are partly in line with previous studies on other sports. Kemper et al. (2015) showed that anthropometric characteristics such as rapid growth rates and BMI increase might help to identify youth athletes at high injury risk, particularly between the year before peak height velocity (PHV) and the year of PHV (DiFiori et al., 2014; Van der Sluis et al., 2015). Additionally, Caine et al. (2014) revealed that the adolescent growth spurt is a time of increased risk for sports injuries; an association between PHV and peak fracture rate was found. Furthermore, the results of Van der Sluis et al. (2015) are in line with those of the present study; the study showed that late maturing soccer players (3.53 injuries/1,000 h of exposure) had a significantly higher overuse injury incidence than earlier maturing athletes (0.49 overuse injuries/1,000 h of exposure), both in the year before PHV and the year of PHV. Moreover, Le Gall et al. (2007) found late maturing athletes at a higher risk of severe injuries and of osteochondral disorders. However, these findings and those of the present study are not in line with the study of Johnson et al. (2009), in which early maturing soccer players showed more injuries than late or normal maturing athletes. It should be noted that in this study, biological age was assessed using X-rays of the left wrist; consequently, actual biological age was calculated (Johnson et al., 2009). Growth timing and diverse proportions were not considered. Additionally, in this study, schoolboy football players were investigated; thus, it might be assumed that they were not top-level youth athletes undergoing a strict selection procedure, as in the present study of youth ski racers, which might explain the differences. Nevertheless, Johnson et al. (2009) emphasized that maturity plus training and playing hours can predict injuries in youth footballers, and consequently, biological maturity status affects the injury risk in young athletes. Jayanthi et al. (2015) additionally found that growth plate tissue may be more vulnerable to injury during periods of rapid growth; consequently, athletes are more susceptible to injuries during more rapid phases of growth. Therefore, it appears particularly important to consider biological maturity status and growth rates in talent development processes to prevent injuries in the future. Additionally, training processes (including volume and intensity) should be commensurate with maturity to prevent late maturing athletes in particular from sustaining injuries.

### Physical fitness parameters as injury risk factors in youth ski racing

To guarantee the optimal development of talented children into elite athletes, physical fitness is an important component of youth athletic performance (Granacher et al., 2016). To date, limited research has examined the link between physical fitness and injury risk in youth ski racing.

In the present study, most of the athletes were either average or above average relative to the graduation of the norm groups in physical performance tests (Raschner et al., 2013). Thus, the youth athletes already had well-developed physical skills; consequently, the athletes could be considered high-level youth ski racers because Raschner et al. (2008) clearly underlined the necessity of a high level of physical fitness to be successful in ski racing, as well as to be able to cope with the demanding requirements during racing. Although the etiology of sport-specific injuries is multi-factorial, it is important to analyze modifiable risk factors associated with a higher injury risk and severity (Chalmers et al., 2013). Several fitness parameters were included in the present study. Results showed that only small percentages of the athletes who were above average with respect to counter movement jump, drop jump reactive strength index, relative core flexion strength, and jump coordination test had a traumatic and/or overuse injury compared with athletes who were average or below average. Only one-fourth of the athletes who were above average with respect to drop jump reactive strength index had an injury (traumatic and/or overuse). These findings confirm the results of the regression analyses, in which drop jump reactive strength index was a significant predictor of both the occurrence of injury and injury severity. The association of drop jump reactive strength index to injury occurrence was the third highest (Wald = 6.146) in the binary logistic model. The multiple linear regression analyses revealed drop jump reactive strength index having the greatest association to injury severity among the identified significant predictors (ß = −1.092). Additionally, significant differences in drop jump reactive strength index were observed between injured and non-injured athletes and between the three groups of injury severity. Non-injured athletes and athletes with mild-minimal or moderate injury severity (in mean) showed better drop jump reactive strength index values. Consequently, within the context of injury prevention in youth ski racing, drop jump reactive strength index seems to be the most predictable factor and should be especially considered in the future.

The drop jump test represents stretch-shortening cycle muscle loading and allows for conclusions to be made about the neuromuscular ability of an athlete. Several studies have shown that deficient neuromuscular control can increase the risk of injuries in youth athletes (Fort-Vanmeerhaeghe et al., 2016). In the present study, athletes with a longer drop jump contact time were at a higher injury risk and were more likely to miss more days of training due to injury. A longer drop jump contact time often accompanies increased knee valgus loading, which is viewed as an injury risk factor or predictor of knee injuries, particularly in female athletes (Hewett et al., 2005). In alpine ski racing, increased knee valgus loading often leads to neurophysiological injuries (Spörri et al., 2017). Three mechanisms were identified for ACL injuries, among which the dynamic snowplow and the slip catch can be partly attributed to increased knee valgus loading (Bere et al., 2011; Jordan et al., 2017). In the present study, only one male athlete sustained an ACL injury; however, the knee was the most affected body part by traumatic and overuse injuries, and ligament injuries in the knee frequently occurred. The trainability of neuromuscular control is highest in preadolescent athletes (Myer et al., 2011). Consequently, neuromuscular training in young athletes can have a preventative effect in reducing the risk of lower extremity injuries (Hewett et al., 1999; Myer et al., 2005, 2009) and should be regularly implemented in training. During alpine ski racing in particular, the knee is exposed to high moments and external forces acting in different directions. Athletes with below-average drop jump reactive strength index, which can result in inappropriate activation timing, may expose their bodies to harmful loads, which induce a higher risk of severe injuries, as was found in the present study. Consequently, the present findings clearly demonstrate that it is absolutely necessary to incorporate neuromuscular training in the athletic-specific training sessions of youth ski racers, yet. The most effective components of neuromuscular training are not well established, or contrasting results have been obtained. Furthermore, research has focused on team sports, whereas to date, individual sports such as alpine ski racing have been examined less or not at all (Emery et al., 2015).

Next to neuromuscular control, sufficient core strength has been proven to be effective in preventing both traumatic (Raschner et al., 2012) and overuse injuries in alpine ski racing (Spörri et al., 2015). The results of the present study showed that among the athletes who were above average with respect to relative core flexion strength, only 20% had a traumatic injury and 30% had a general injury. The logistic regression model confirmed the finding that young ski racers with better relative core flexion strength were at a lower total injury risk. Moreover, athletes with a higher relative flexion/extension strength ratio were more likely to miss more days of training due to injuries.

Trunk strength is considered to be important in compensating for external forces and loads during alpine ski racing (Spörri et al., 2015). Zazulak et al. (2007) demonstrated a relationship between reduced muscular trunk strength and an increased knee injury risk in young athletes. Knowing that alpine ski racing is a high-risk sport for knee injuries, as it was also shown in the present study, it is crucial to monitor core strength in youth athletes, particularly because the results of Raschner et al. (2012) showed that core strength weakness and core strength imbalances were significant risk factors for ACL injuries in adolescent ski racers. The authors emphasized that coaches must understand the importance of core strength training, and especially the neuromuscular aspects of core training, in the context of injury prevention in youth athletes (Raschner et al., 2012). Because of the importance of well-developed core strength for coping with the unexpected perturbations during racing, a focus should be placed on diverse training contents activating differing reflex activity patterns of the trunk muscles. In this context, Mueller et al. (2016) suggested to use lower leg perturbations of greater magnitude to evaluate the neuromuscular reflex activity patterns of the trunk muscles. Additionally, Pedersen et al. (2004) argued that in injury prevention programs, perturbations should be implemented with increasing frequency to enhance the level of difficulty of sensorimotor trunk stability exercises and to prepare athletes for compensation of unexpected trunk loading, which seems very important in alpine ski racing. Considering this, next to conventional balance training contents to improve core strength, slackline training could be considered in training sessions of youth ski racers because of the high perturbations and the eventually associated preventative effect, even though statistical evidence has not been found as of yet (Granacher et al., 2010; Keller et al., 2012). However, Keller et al. (2012), underlined the motivational aspect of slackline training compared with classical balance training, because for athletes slacklining may be more joyful and even more demanding. Nevertheless, further research is necessary to prove the preventative effect of slackline training in young athletes (Granacher et al., 2010; Keller et al., 2012). In general, Taube et al. (2008) and Hübscher et al. (2010) emphasized the beneficial effect of diverse balance training contents for injury prevention; it was shown that sensomotoric training leads to an increased motoric control of the musculature responsible for knee and ankle joint stabilization (Gollhofer et al., 2006; Taube et al., 2006), which might be beneficial for a lower injury risk. While all these findings lead to the assumption that neuromuscular core strength training might contribute to the decrease of injury risk in youth ski racing, further research is necessary.

The findings of the present study confirm that there appears to be no relationship between endurance capacity, leg strength and injury risk. At this point, it is important to mention that for coaches, the aim of physical testing is not to make appropriate decisions associated with the future success of their athletes. From a functional point of view, it is more important to discover individual weaknesses and imbalances among young athletes. Results should be used to develop individualized training programs, based on maturational characteristics if necessary, to help prevent injury.

### Injury rate and severity

In general, smaller rates of traumatic injuries and overuse injuries were found in the present study compared with athletes competing at the adolescent level (1.7 injuries/1,000 h ski training) (Westin et al., 2013) and those competing at the elite level (36.7 injuries/100 athletes) (Flørenes et al., 2009; Bere et al., 2013; Haaland et al., 2016; Spörri et al., 2017). Fort-Vanmeerhaeghe et al. (2016) explained that prepubescent athletes may be at lower injury risk because they have lower body mass and shorter joint lever arms and because they do not generate as much dynamic valgus load as their more mature counterparts. Additionally, most injuries were classified as moderate based on an absence from training between 8 and 28 days. These findings are in line with the results of Hildebrandt and Raschner (2013) among 15- to 19-year old ski racers. In contrast, at the World Cup level, most injuries have been classified as severe (35.6%) (Bere et al., 2013). However, the retrospective study design employed in both studies (Bere et al., 2013; Hildebrandt and Raschner, 2013) should be considered a limitation. It is possible that minimal and moderate injuries were not assessed because of missing data, providing one reason why the results at the World Cup level differ from the results at the youth level.

## Limitations

The diverse sample sizes based on the norm group classification and the three maturity groups must be considered a limitation of the study because direct comparisons are difficult. Furthermore, the rates of injuries were relatively low during the study period. Therefore, in some sub-categories, there were too few cases to yield conclusive results. Further investigations with larger sample sizes and a longer observation period are recommended to detect risk factors between groups. Another limitation of the study was that the potential changes in different parameters (such as changes in height and weight, muscle mass and performance) from the start of the study to the end of the study were not considered as possible risk factors. Within the scope of this study, it was not possible to analyze training-related parameters. Previous research has shown that relative increase of training volume is one of the most important predictors of injuries (Gabbett, 2016; Soligard et al., 2016); therefore, the quantification of training load, variations during the year and the risk of injuries should also be investigated based on a daily monitoring system. Results of the drop jump test were only based on quantitative values. However, a video analysis can provide additional information about valgus lower limb alignment.

## Conclusion

Although there is no single fitness parameter responsible for determining the risk of injuries, a comprehensive fitness regimen starting at a young age is crucial for coping with the physical requirements of alpine ski racing and minimizing the rate of both traumatic and overuse injuries. The findings of the present study showed that decreased core strength and below-average drop jump ability are accompanied by a higher risk of sustaining a severe injury. DJ RSI was a significant predictor of the occurrence of injury and injury severity. This variable significantly differed between injured and non-injured athletes, as well as between injury severity groups. With respect to both regression models, the DJ RSI showed the greatest (injury severity) or third highest association (occurrence of injury). Thus, these findings underline the importance of neuromuscular training in youth athletes in the context of injury prevention. Additionally, anthropometric characteristics and biological maturity status significantly affected injury risk in youth ski racers. It has to be considered that the two regression analyses could explain only 45–47% of the variability of the athletes and therefore, more than half of the variability remains unexplained. However, Spörri et al. (2017) emphasized the multifactorial nature of injury causes in a changing outdoor environment, which decreases the chance of identifying statistically significant risk factors. In this context, the explained variabilities of the present study seem to be high because nearly half of the variability could be explained by the investigated variables, which seems important for future injury prevention in youth ski racers. However, in general, the occurrence of injuries was relatively low compared with that of elite alpine ski racers. Therefore, the regression analyses were performed for traumatic and overuse injuries combined. In the future, risk factors should be assessed separately for both types of injuries. Nevertheless, a regular, long-term documentation of changes in anthropometrics, physical fitness and training loads is important to quantify the effect of risk factors on alpine ski racing-specific injuries. Additionally, Fort-Vanmeerhaeghe et al. (2016) emphasized that youth athletes may need longer recovery phases than adults, which also seems important among youth ski racers. Consequently, recovery phases and regeneration measures must be implemented in the training process of young ski racers in order to possibly contribute to the prevention of injuries, particularly of overuse injuries, from occurring.

Based on studies in other types of sport, future research should evaluate the role of leg dominance (Smith et al., 2015) as a potential and modifiable risk factor in youth ski racing and the effects of diverse components of neuromuscular training on injury risk reduction (Emery et al., 2015). All analyses should contribute to injury prevention in young athletes because sports injuries in youth can become a barrier to long-term physical activity and can prevent athletes from enjoying successful careers (Emery et al., 2006, 2015; Caine et al., 2014).

## Author contributions

All authors listed have made substantial, direct and intellectual contributions to the work and have approved it for publication.

### Conflict of interest statement

The authors declare that the research was conducted in the absence of any commercial or financial relationships that could be construed as a potential conflict of interest.
